# Optimizing Correction Factors on Color Differences for Automotive Painting Services

**DOI:** 10.3390/s24248213

**Published:** 2024-12-23

**Authors:** Emilia Corina Corbu, Anne-Marie Nitescu, Eduard Edelhauser

**Affiliations:** 1 Department of Mathematics and Informatics, University of Petrosani, 332003 Petrosani, Romania; corinacorbu@upet.ro (E.C.C.); annemarienitescu@upet.ro (A.-M.N.); 2 Department of Management and Industrial Engineering, University of Petrosani, 332003 Petrosani, Romania

**Keywords:** color difference, sensory analysis, human visual perception, image analysis, CIELAB, optimization, sensor devices

## Abstract

Currently, the automotive sector is showing increased demands regarding the color of cars in general, but especially the quality and the time of painting, in particular. Companies working in this industry, especially in specialized painting services, must perform work of impeccable quality in the shortest possible time in order to be efficient. Color differences that appear in different areas of the car result from the use of different formulas for obtaining color. These differences can be reduced by using correction factors that are established for the colors in the partial or total painting process of cars. There are several factors that lead to settings that are not verified by the real color and, therefore, contribute to incorrect color results and also to high and unnecessary repair costs. In this study, the authors aimed to optimize the values of the correction factors applicable in the automotive industry, based on a set of 135 measurements performed with a BYK Gardner spectrophotometer, in order to minimize color differences. Through this study, authors have also aimed to find out how the color-identification process can be streamlined with the smallest possible tolerances by optimally adjusting the correction factors and by identifying the factors that influence the color-reading and identification process. A total of 85 pairs of samples were used for the DS1 (data set) and 53 pairs of samples for the DS2 (data set); these samples were used in the visual experiments for testing the performance of two color-differentiation formulas. The first part of the research aimed to investigate the visual perception of the painted cars in terms of differences in brightness, chroma and hue, data that were used to optimize the formulas used for color differences. Finally, authors have estimated the closest color variant to the objective color by optimizing the correction factors and thus achieving the efficiency of the color-identification process and the whole painting-identification process.

## 1. Introduction

Visual appearance and color play a decisive role in industries such as luxury goods or the automotive industry. New colors and new trends in the automotive sector have led to the idea of a new revolution in coating [1].

In the luxury automotive industry in particular, whatever the cause, sale or accident, a vehicle may require a repair of a specific element or of a certain area, a repair that must be undetectable and invisible to the customer, depending on the color formula used. Regardless of the techniques used in the repair process, a color difference will be observed between the original color and the formula proposed by the colorimeter. These color differences create various problems: the differences persist if the paint is not sprayed on adjacent elements, they increase the time for color identification, or they lead to material waste if a color with an exaggerated tolerance has been chosen.

Lighting conditions, more precisely, quantifying the light sources and establishing the same reference for paint manufacturers such as BASF [2] or Cromax [3], provide color formulas that allow the reproduction of every color shade of the automotive fleet, which includes several hundred thousand colors. Solid, or effect, colors and metallic, or pearlescent, colors are obtained from ingredients mixed together. Depending on the angle at which they are viewed, light produces different effects on the paint that can modify the lightness and/or hue of the color itself. The effects are responsible for the heterogeneity of the behavior of the optical film and, therefore, the effect, that is, the texture.

Quality programs have imposed the replacement of visual color QC with instrumental evaluation, partially eliminating human subjectivity [4]. In order to ensure a balance between instrument-based QC and human perception, spectrophotometers have been introduced by major companies in the automotive industry [5].

There are differences between the paint used by paint systems in automotive factories, what is used on metal parts, and that used on plastic parts in workshops. In addition, the paint, applied in layers, has components with different melting temperatures that lead to different temperatures for paint application and drying [6]. Also, temperature and humidity can differ from one supplier to another and lead to color differences between automotive parts [7]. Color perception represents the transformation of light into color; the visible range for the human eye is between 380 nm and 780 nm [8]. The color of the spectrum changes with temperature [9,10]. Based on experiments, a standardized system, the CIE Standard Colorimetric Observatory, was adopted in 1931 [11]. Now CIELAB represent coordinates L*, a*, b*, calculated according to ISO/CIE 11664-6:2022, and for use in the paint and coatings industry was released a tolerance method by CIE, called CIE 94 [12], and also for colorimetric calculation of color differences it is used DIN 99 [13].

The present study analyzes the color-control issue faced by the majority of operators in the auto repair and paint sector and, based on the measurements performed, the authors have tried to propose solutions by estimating the value of correction factors to minimize color distances, depending on the instrumental readings taken with a BYK Gardner spectrophotometer.

## 2. Materials and Methods

The study objective was to determine the correction factors for color matching, which take into account the specific particularities for the automotive industry in concrete working conditions specific to an area, such as temperature and humidity. The study objective is very important because the correction factors presented in the calculation formulas in the literature are determined for theoretical conditions, but this study was focused on obtaining a color difference tending to zero in practical conditions.

In car services, such as those the authors have investigated, the painting process must be completed in a contracted time. The color differences which result from the painting, which the authors have quantified in deviations, or color differences, had multiple causes and brought delays in the completion time of the work, as well as a loss of money if errors are made in the painting process. That is why the authors considered that the speed of identifying the correction factors for a certain situation can reduce the loss of time and money in the repair process. That is why the authors called this process optimizing the correction factors adapted to the working conditions and not to a reference condition, or theoretical condition.

### 2.1. Data Collection

**Figure 1:**
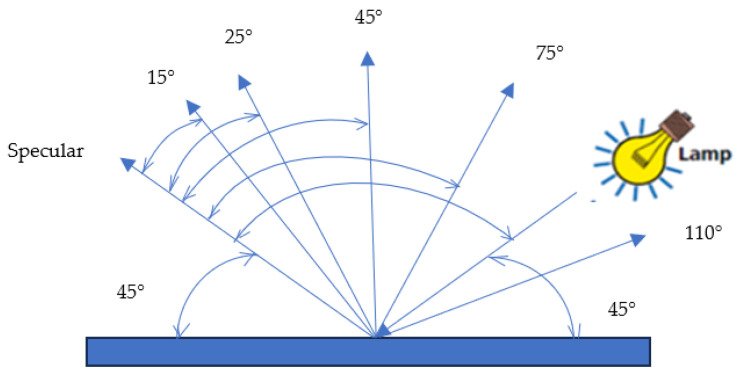
Color-measurement geometries for the BYK Mac (−15 is not drawn) from BYK Gardner GMBH, 2009 [14].

Data were collected using a BYK Gardner spectrophotometer, which is a multi-angle spectrophotometer equipped with a camera, and the authors used an illumination system placed at 45° to the normal, as well as 6 detectors set at −15°, 15°, 25°, 45°, 75° and 110°, presented in Figure 1.

All measurements were performed by the authors under the working conditions of an authorized car service. Instrumental measurements were performed under DN65 illumination conditions with a BYK Gardner model 6320 spectrophotometer. The BYK Gardner spectrophotometer was chosen as the measuring instrument because it is a spectrophotometer commonly used in Italian services, and the authors followed the processes carried out in ordinary car services. Technical and standard data are as follows: temperatures range from 10 °C to +60 °C (14 °F to 140 °F); humidity measurements for operation are up to 85% non-condensing/35 °C (95 °F); geometry measurement illumination 45°; observation 15°, 45°,110° [14].

Data sets are represented by pairs of samples with color differences ranging ∆E_ab_ between 0 and 10, with the color differences not scalable over such a large range [14]. Color-difference equations are performed for very small color values (∆E_ab_ < 1), leading to the region of interest for QC applications [15,16].

Approximately 135 readings were taken between June 2023 and October 2024. These readings were grouped into two sets of measurements: data set DS1 and data set 2 DS2.2.

**Figure 2:**
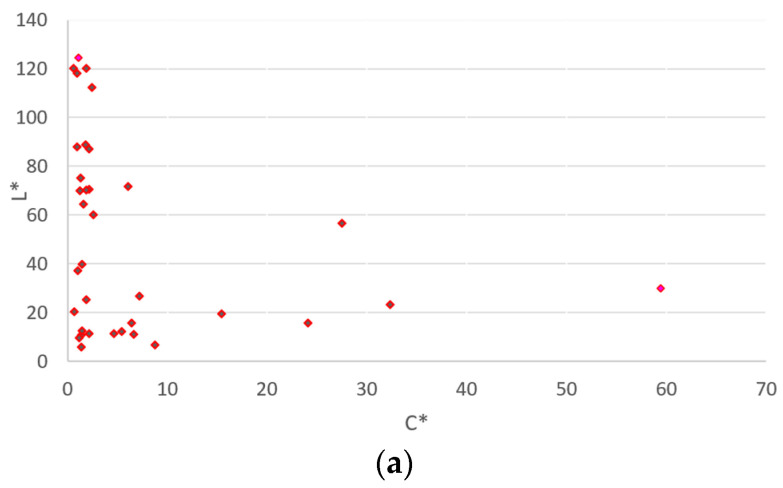

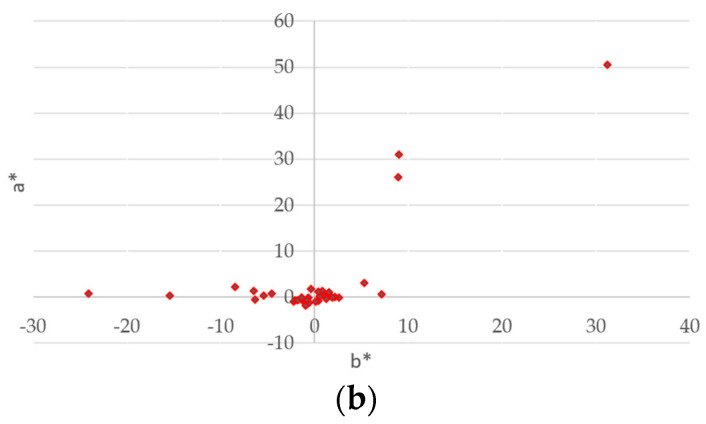
(**a**). DS1 48–82 Color distribution on lightness scale L_ab_* and chroma C_ab_*; (**b**) DS1 48–82 color distribution on green–red scale—a*—and blue–yellow scale—b*— respectively.

**Data set DS1**. Approximately 85 simple readings were taken on vehicles or individual parts. For each color reading, the authors identified the CIELAB coordinates read by the spectrophotometer at angles of 15°, 45° and 110°. In Figure 2, 48 points are readings with a spectrophotometer from data set 1—DS1, and 82 are readings with a spectrophotometer from data set 1—DS1.

For 35 simple readings from DS1, authors have represented the color distribution in Figure 2a, which shows coordinates on an a*(green–red)–b* (yellow–blue) scale in CIELAB units -, in Figure 2b. a* in CIELAB is the green–red axis. A positive value of a* means that the respective color tends to red and a negative value tends to green. From these 85 simple readings, authors have plotted the readings labeled 48, 49, 50, 51, 51, … 60, 61, 62, …, 70, 71, 72, … 80, 81 and 82 in Figure 2, and these are the 35 represented data.

**Figure 3:**
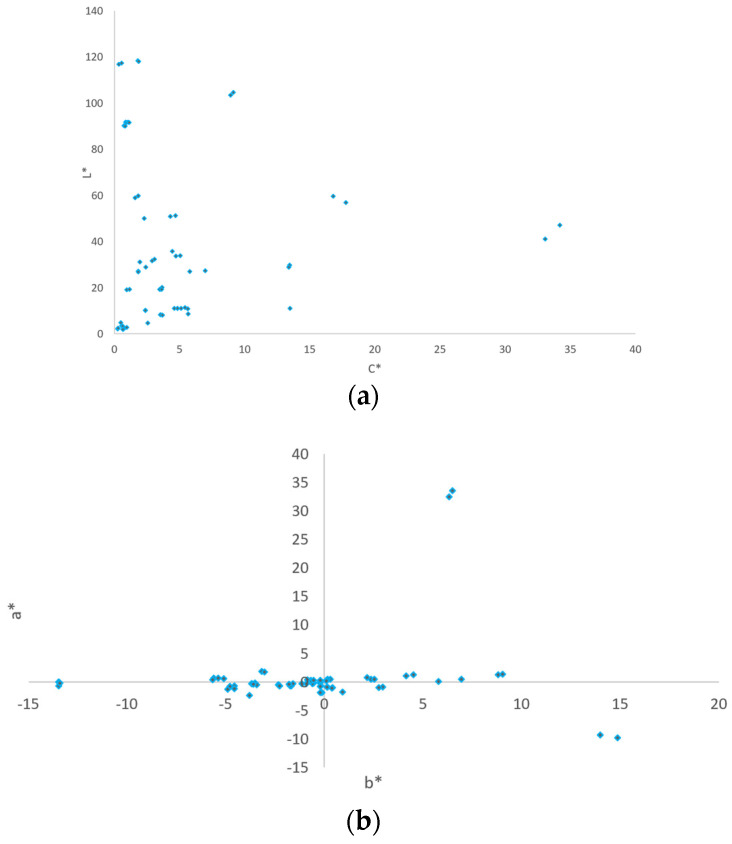
(**a**). DS2 46–151 color distribution on lightness scale L_ab_* and chroma C_ab_*; (**b**) DS2 46–151 color distribution—a* b*.

**Data Set DS2.** Approximately 35 instrumental readings were performed with two or three readings (samples) on different components of the same vehicle. For each vehicle in the service ready to be painted, in the phase of identifying the color to be prepared by the colorimeter, authors performed two or three readings (samples) on the components to be painted. These data constitute data set DS2 and are represented in Figure 3a,b.

In this first part of the research, authors have investigated the color differences for each color sample for pairs of measurements that refer to lightness differences ∆Lab*, chroma differences ∆C_ab_*, or hue differences ∆H_ab_. Thus, there were 52 pairs tested because this represented the number of pairs with both chroma parameters bigger than value 5 (C_1_* > 5 and C_2_* > 5). 

### 2.2. Color Distances in Standards CIELAB, DIN99 and CIE94

Authors have represented the visual evaluation using three parameters, lightness, chroma (saturation) and hue, in a 3D diagram. Authors have also used the variable lightness (ΔL) to quantify the differential luminance threshold for eye-sensitivity detection. [15].

Since 1976, the CIELAB color space has been based on a nonlinear transformation of the CIE tristimulus coordinate color space X, Y, Z. CIELAB is uniform (the calculated distances between samples must correlate uniformly with the visual differences between the samples) and simple (it must provide a simple means for the user to interpret the data).

**Figure 4:**
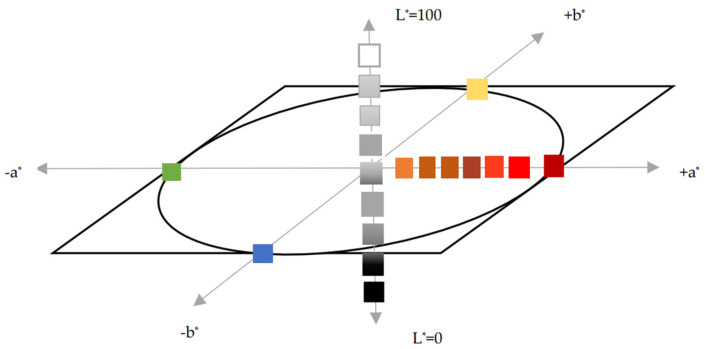
Color space CIELAB.

The CIELAB color space, presented in Figure 4, is a uniform and device-independent scale where each perceived color is defined by a color point that has the coordinates {L*, a*, b*}. Green and red lie on the a* axis, and the b* axis corresponds to the opposing colors blue and yellow. The L* axis, the neutral gray axis, is perpendicular to this plane and represents lightness. Its endpoints are black (L* = 0) and white (L* = 100), and the intermediate values on this axis are achromatic shades of gray.

CIELAB makes it possible to define color differences. Numerous computational models have been developed to better represent color differences that can be distinguished by the human visual system, a non-exhaustive list being CIE76, CIE94, CMC, CIE 2000, AUDI95 and AUDI2000 [16,17,18]. Luo and Cui [17] identified the generic equation for calculating the color difference in CIE2000.

The color differences measured in CIELAB color space and ∆L_ab_*, ∆a*, and ∆b* have the advantage of being relatively simple. The authors have used Formula (1) to calculate ∆E_ab_*:

(1) $${{\Delta E}_{ab}}^{*}=\sqrt{{\left(\Delta L_{ab}^{*}\right)}^{2}+{\left({\Delta a}^{*}\right)}^{2}+{\left({\Delta b}^{*}\right)}^{2}}$$

The calculated chromatic distances do not correspond to the perceived chromatic distances for all colors. In practice, this means that for achromatic colors, the human eye can distinguish the smallest differences in color hue.

(2) $${{\Delta E}_{ab}}^{*}=\sqrt{{\left(\Delta L_{ab}^{*}\right)}^{2}+{\left(\Delta C_{ab}^{*}\right)}^{2}+{\left(\Delta H_{ab}^{*}\right)}^{2}}$$

where ∆L_ab_*, ∆C_ab_*, ∆H_ab_* are the CIELAB metric differences of lightness, chroma and hue.

The basis for the DIN99 [19,20,21] color space is the CIELAB color space with its L*, a*, and b* coordinates. The brightness in DIN99 was noted by L_99_ and calculated with the following equation:

(3) $$L_{99}=\left(\frac{1}{k_{E}}\right)\cdot \left(105.51\cdot ln\left(1+0.0158\cdot L^{*}\right)\right)$$

The individual calculations are as follows: a* and b* are transformed to e and f.

Redness value (red/green axis) e is calculated with (4):

(4) $$e=\left(a^{*}\cdot cos\left(16^{{}^{\circ}}\right)+b^{*}\cdot sin\left(16^{{}^{\circ}}\right)\right)$$

Yellowness value (yellow/blue axis) f is calculated with (5):

(5) $$f=0.7\cdot \left(-a^{*}\cdot sin\left(16^{{}^{\circ}}\right)+b^{*}\cdot cos\left(16^{{}^{\circ}}\right)\right)$$

From this, the chroma value G (colorfulness) is then calculated:

(6) $$G=\sqrt{\left(e^{2}+f^{2}\right)}$$

In addition, the compression factor k is calculated:

(7) $$k=\frac{ln\left(1+0.045\cdot G\right)}{\left(k_{CH}\cdot k_{E}\cdot 0.045\right)}$$

The variable k_E_ describes the influence of changed observation conditions. Under reference conditions, k_E_ = 1.

Value a99 in DIN99 is given by the ratio between e and G multiplied by the compression factor k, and b99 is given by the ratio between f and G multiplied by the compression factor k.

∆E_99_ was calculated with:

(8) $$\Delta E_{99}=\sqrt{{\left({\Delta L}_{99}\right)}^{2}+{\left({\Delta a}_{99}\right)}^{2}+{\left({\Delta b}_{99}\right)}^{2}}$$

In the case that a* = b* = 0, also e = f = G = 0, then a_99_ = b_99_ = 0

The formula for determining color distance in CIE94 [19,20] is as follows:

(9) $$\Delta E_{94}=\sqrt{{\left(\frac{\Delta L^{*}}{k_{L}S_{L}}\right)}^{2}+{\left(\frac{\Delta C^{*}}{k_{C}S_{C}}\right)}^{2}+{\left(\frac{\Delta H^{*}}{k_{H}S_{H}}\right)}^{2}}$$

where: 

Lightness deviation is the difference between the lightness of the two readings L_2_* and L_1_*. Chroma deviation is the difference between the chroma of the two readings C_2_* and C_2_*. Hue deviation is the difference between the hue of the two readings H_2_* and H_1_*. The value of S_L_ is 1.

(10) $$S_{C}=1+0.045C^{*}$$

(11) $$S_{H}=1+0.0015C^{*}$$

Therefore, the lowest possible numerical values of ∆E*ab had to be determined. The brighter the evaluated color shades, i.e., the higher the C* values, the further apart the colors are in the CIELAB system and the lower the sensitivity with which the human eye reacts to color distances [22,23].

**Figure 5:**
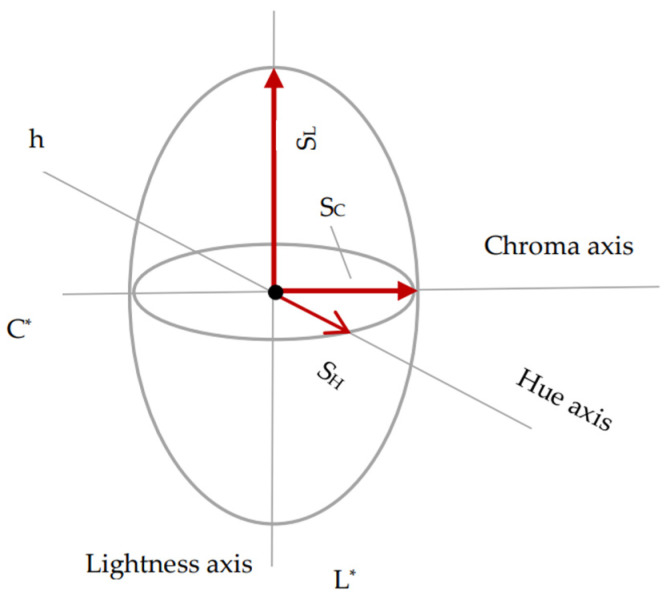
Color space CIE94.

The authors’ study is based on the differences resulting from the CIELAB, DIN99 and CIE94 spaces. DIN99 is adopted by the German DIN standardization institute and used by the German automotive industry [24]. The second space considered in this study was CIE94, presented in Figure 5.

Independent tests of the DIN99 modification show a performance comparable to the best of the other color-difference equations [20,25,26,27,28]. Also, there are no differences between ∆E_cmc_ in CMC and ∆E_00_ CIEDE2000 or ∆E_94_ CIE94 [29,30,31,32,33,34,35]. Only, Cui and Luo [26] presented several modifications of the DIN99 space, improving the description of color differences.

### 2.3. Visual Differences and Optimization Methods

Large-scale performance tests of color-difference equations applied to glossy paint samples with relatively small color differences are based on the so-called RIT-DuPont data sets [36] and Leeds [37]. These data sets contain only solid colors, and therefore did not include samples that could correspond to the darkest or lightest metallic colors. 

He [38] quantified the visual color difference (∆V) measured by the STRESS (Standardized Residual Sum of Squares) index, and Garcia [39] proposed the use of the STRESS index:

(12) $$STRESS=100\sqrt{\left(\frac{\sum {\left(∆E_{i}-f∆V_{i}\right)}^{2}}{\sum ∆E_{i}^{2}}\right)}$$

where

(13) $$f=\frac{\sum \Delta E_{i}\Delta V_{i}}{\sum V_{i}^{2}}$$

where ∆L_ab_*, ∆E_ab_*, ∆C_ab_*, and ∆H_ab_* are the CIELAB metric differences of lightness, chroma and hue.

In the CIE94 formula, k_L_, k_C_, and k_H_ are three correction factors. S_L_, S_C_, and S_H_ are three weighting functions for the differences of luminance, chroma and hue. k_L_, k_C_, k_H_ and S_L_, S_C_, S_H_ were all set to 1. In CIE94, the color difference was calculated with Equation (9) and S_L_ = 1, S_C_ and S_H_ are two weighting functions specified by Equations (10) and (11).

Considering that these correction coefficients were considered as reference conditions for many experiments, that is, they had values of 1 and for certain industries like the textile one, the value k_L_ = 2, k_C_ = k_H_ = 1 was given, the authors set out to optimize the value of one of these correction factors, namely k_L_.

Authors have improved the predictions of the CIELAB, DIN99 and CIE94 color difference formulas using an optimization method, so k_L_ was optimized with k_C_ = k_H_ = 1. The goal of this study and optimization was to minimize the STRESS value between the visual results and the values calculated using a color-difference formula using the Generalized Reduced Gradient (GRG) method in a Solver application. The Generalized Reduced Gradient (GRG) method is an extension of the Reduced Gradient method to accommodate nonlinear inequality constraints. In this method, a search direction is found such that for any small move, the current active constraints remain precisely active.

### 2.4. Research Methodology 

Each vehicle enters in a service with a Technical Data Sheet containing the following information: manufacturer, model, year of manufacture, color code and component that requires repair. For each vehicle in the Technical Data Sheet there is a mandatory Factory Color Code.

The readings in our study were made with a spectrophotometer, Cromax, in order to identify the color with which the analyzed piece is painted. Two or three readings are made on the respective pieces; that is why the authors called them pairs of readings. The readings are expressed in Lab coordinates. The differences between these coordinates were marked with ∆L and the color differences in Lab with ∆E_ab_.

The Factory Color Code is taken into the colorimetric program, which breaks down the Color Code into basic colors and the pigments that make up that color. 

It is visually noted that there are very visible color differences between the color identified by the Factory Color Code and the color identified by the spectrophotometer. The explanation for these differences is the fact that components of the vehicle or even the entire vehicle have undergone previous repairs and, implicitly, paints that have changed the color or endured solar, thermal, water, wear and tear effects.

For the noticeable visual differences at this stage, there are several causes, respectively: (1) The distance and position of the paint gun in relation to the part; (2) The speed with which the painting is done; (3) Adjusting the paint gun; (4) Loading with dyeing product; (5) Force drying; (6) Temperature and air flow and humidity (factors influencing color variation).

**Figure 6:**
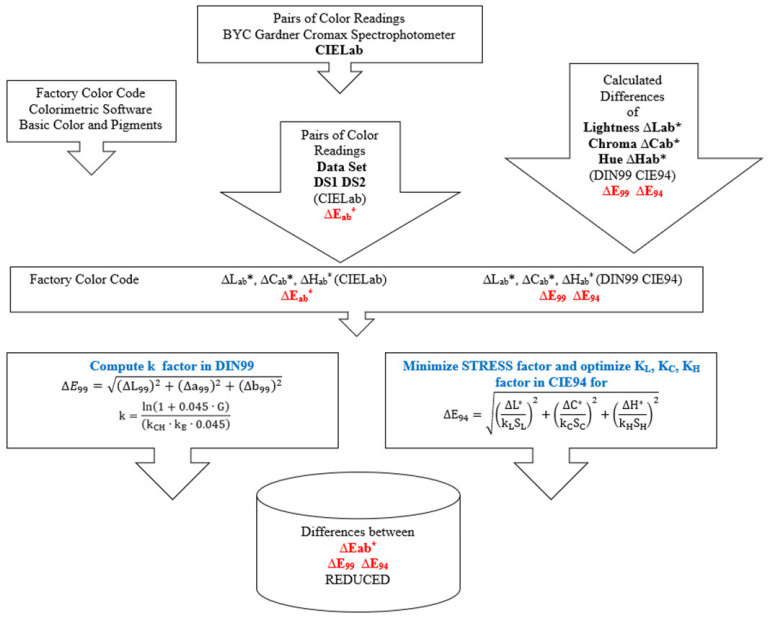
Conceptual research model.

Based on the previously presented aspects, the authors have set a conceptual research model, used in the current study, as it is presented in Figure 6, where k factors (k., k_L_, k_C_ and k_H_) marked in blue represent optimized corrected factors, and the distances marked in red ∆E_ab*_, ∆E_99_ and ∆E_94_, represent differences calculated with formulas.

## 3. Results

### 3.1. Experimental Conditions

The results of this study were obtained under the experimental conditions presented in Table 1—color, date, temperature, humidity—and for the types of paint, such as presented in Table 2—car model, color name and Color Code.

### 3.2. Results of Instrumental Monitoring

The CIE [40] recommended measuring object colors in the CIELAB color space, and the associated CIELAB color difference formula has been widely used to quantify the perceived color difference between a pair of colored samples.

**Figure 7:**
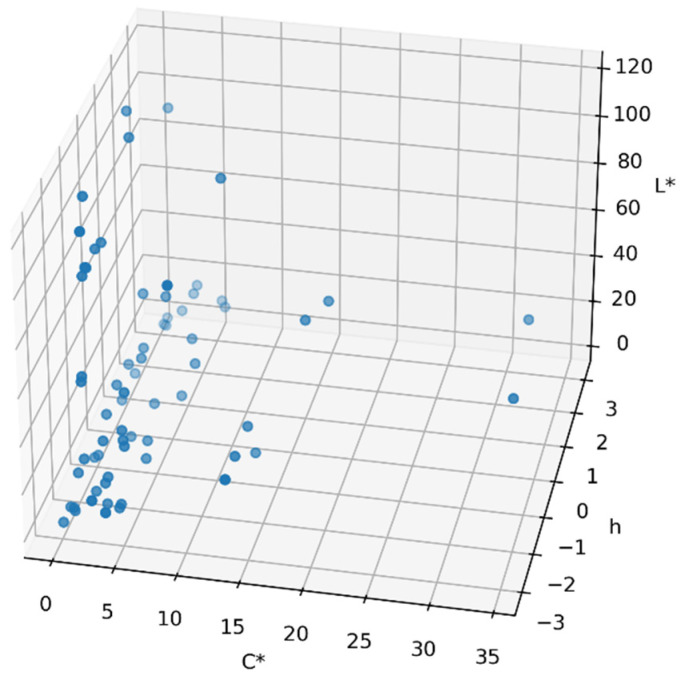
Representation with 3D points for data sets DS1 with the following Cartesian coordinates: (lightness L*, chroma C* and hue h*).

The authors have plotted measurements 48–82 in 3D in Figure 7 to have the representation in a 3D plot, in Spyder IDE (Python 3.1) [41].

Kuehni [42,43] proposed for the evaluation of color deviation to use chroma C*. If chroma is less than 5 (C* ≤ 5), it would be better to use ∆L*, ∆a* and ∆b*, and for chroma C* > 5, then use L*C*h. For evaluation of the color deviation it would be better to use ∆L*, ∆C* and ∆H*. According to the results reported in Figure 6, the authors have observed that the chroma parameters C1*/C2* are < 5 for samples 46–47, 83–84, 88–89, 109–110, 115–116, 117–118, 138–139, 140–143, 141–142, 144–145, and 150–151, so the color deviation can be expressed for these measurements in ∆E_ab_ *. The authors also observed that the parameter ∆E_ab_* remains in the range ∆E_ab_* < 1 for the measurements indicated above. 

**Figure 8:**
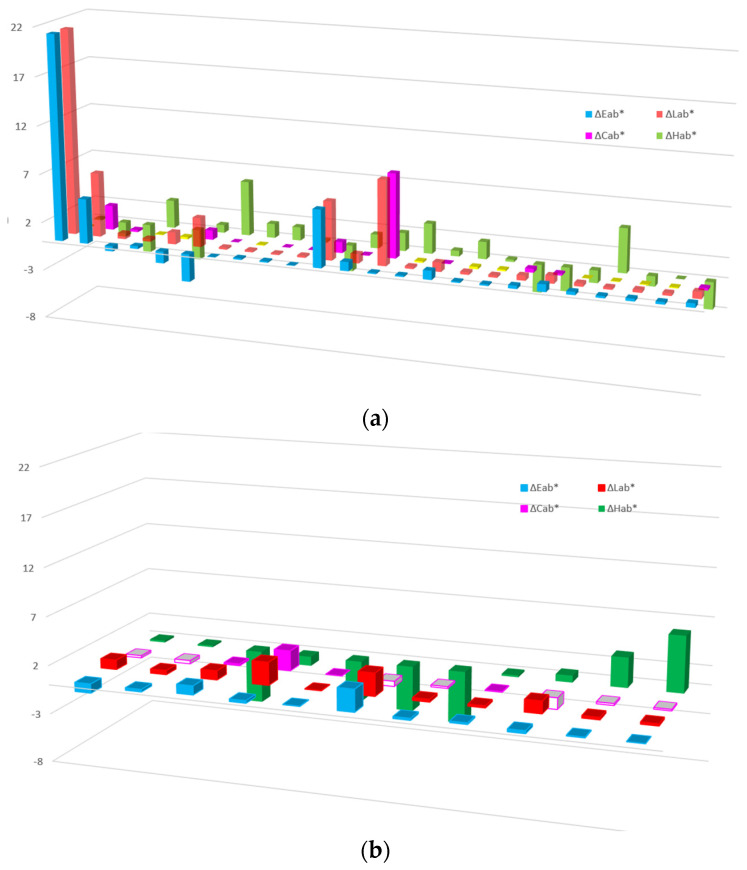
(**a**) Differences in brightness ∆Lab*, in CIELAB ∆Eab*, in chroma ∆Cab*, and in hue ∆Hab* for DS2 46–47 to 141–142; (**b**) Differences in brightness ∆Lab*, in CIELAB ∆Eab*, in chroma ∆Cab*, and in hue ∆Hab* for DS2 for the remaining pairs.

The differences in Figure 8a include the following samples: 46–47, 83–84, 86–87, 88–89, 90–91, 107–108, 109–110, 109–110, 109–110, 110–111, 113–114, 115–116, 115–116, 115–116, 117–118, 117–118, 117–118, 138–139, 138–139, 140–143, 140–143, 140–143, 141–142, and 141–142.

The color differences determined after DS2, expressed in ∆Eab*, are smaller for dark, achromatic colors than for chromatic colors. The smallest visible color difference is approximately ∆Eab* = 0.3 for dark, achromatic colors, increasing to ∆Eab* = 0.7 for bright yellow and red-orange colors, according to [19]. The authors have observed that the differences exist also when ∆Eab* < 0.3, as in the case of measurements 88–89, 109–110, 110–111, 117–118, 140–143, 146–147, and 150–151 in Figure 8a,b. 

### 3.3. Color Differences and Visual Color Difference

Following the method presented in [38], observer variability was quantified using the STRESS index [44]. Melgosa obtained the visual color difference (∆V) values obtained from the grayscale scores reported by the observers participating in the experiments [38]. The conversion of grayscale to ∆V visual difference for each data pair was performed with Equation 14. In Equation 14, the color from the CIELAB color space representation was converted to RGB using Lab2RGB from MATLAB, and then the authors used the function rgb2gray to convert the image to grayscale.

(14) $$\Delta V_{ab,10}=-0.25*GS^{3}+3.05*GS^{2}-13.95*GS+24.46$$

The above equation is for CIELAB. The authors have plotted the color differences ∆E_ab_* as a function of the visual color differences ∆V.

**Figure 9:**
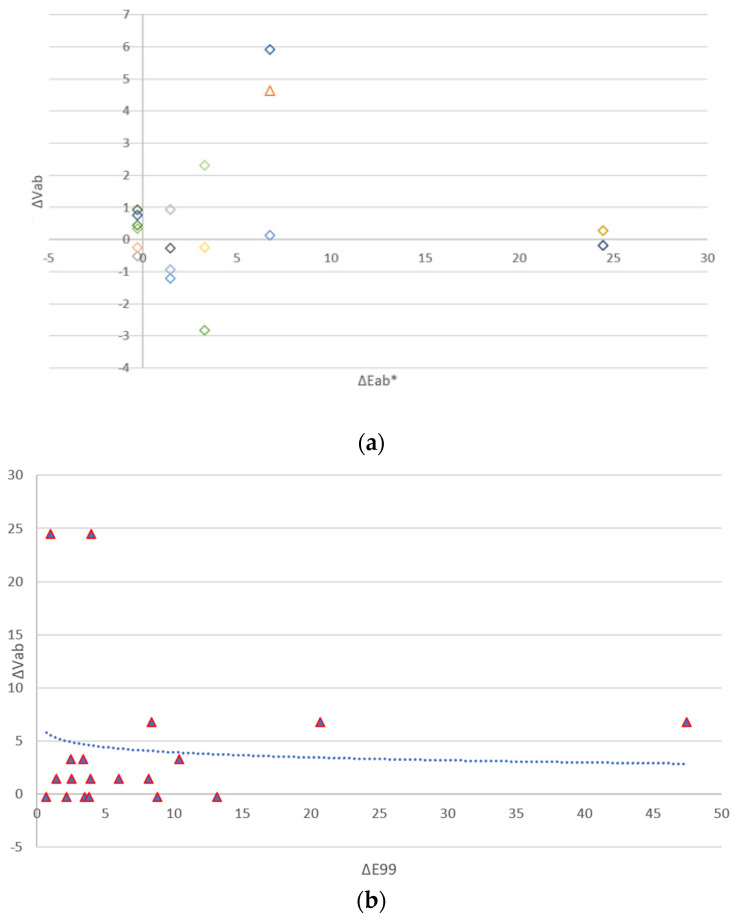
(**a**) Representation of the differences ∆Eab* as a function of ∆V. (**b**) Representation of the differences ∆E99 as a function of ∆V.

The authors have represented 19 pairs of measurements in Figure 9a,b. The blue dash represents a trend line, and the authors have identified the trend line as a logarithmic function:

y = −0.688 ln(x) + 5.5088

### 3.4. Color Distances and Correction Factors in DIN99 and Optimization of Correction Factors in CIE94

The CIEDE2000 color difference formula was developed to improve the correlation between calculated and perceived color differences [17,45]. For measurements in DS2, the authors have used chroma parameter C1* > 5 and C2* > 5, ∆E_99_, and then they have calculated the color differences in the DIN99 and in the CIE94 space ∆E_94_. For the calculation of color distances, authors have used the DIN99 color space, since its application is recommended for small color distances ∆E of up to 5 CIELAB units, such as those addressed in quality assurance and recipe calculation.

The authors have noticed that one of the factors that influenced the measurements in the DS2 set was the curvature of the surfaces. The authors also noticed that on some car models, adjacent parts are not parallel, but have a relatively small angle between them. For effect coatings, this can lead to a small but visible visual difference between these parts. The authors took double or triple readings because the car models on which the measurements were performed have curvatures of the front and rear wings that give different results.

**Figure 10:**
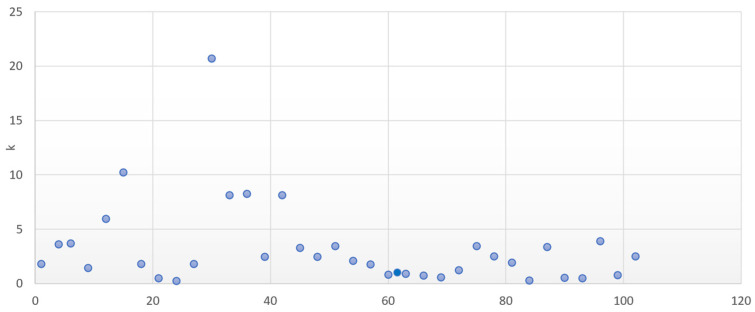
Compression factor k in DIN99.

In the DIN99 space, the resulting mathematical values of the compression factor k are presented in Figure 10 for the DS2 set of sample pairs. The authors have found a compression factor between 1.5 and 3, the additional parts being separated from the adjacent parts of the body by a dark space, and a factor of approximately 1.5 for measurement geometries close to the glare angle.

Other factors that influenced the measurements and, implicitly, the color differences were the temperature and humidity conditions. In an attempt to compensate for these variations, authors have used visual identification, which makes the color differences less critical. Other situations in which the correction factors must be modified are situations in which the visual texture, in effect, coatings or the brightness of the metallic coatings, is very high (L* > 100), as in the case of samples 141–142.

To improve the appearance of car color using the predictions of the CIELAB and CIE94 color difference formulas, authors have used a k_L_ factor-optimization method with k_C_ = k_H_ = 1. The goal of the optimization was to minimize the STRESS value between the visual results, and the values were determined with a color-difference formula using the GRG method in Excel Solver. A k_L_ factor value of 1.0429 was obtained.

### 3.5. Practical Results of the Optimization of Correction Factors in CIE94

Because the authors’ method is tested under practical conditions, such as an automotive painting service, the authors assess its effectiveness in real-world applications. A visual comparison of before-and-after optimization results provides intuitive evidence of the improvements made with the authors’ optimization, as can be seen in Table 3.

- This study has customized and has applied the calculation formulas for the differences in brightness, chroma and hue in two spaces, DIN99 and CIE94, but it was found that the differences calculated in the spaces DIN99 and CIE94 differ from the differences measured in the research made with the spectrophotometer in CIELAB, which is why the optimizations were necessary.
- The differences in the two spaces, DIN99 and CIE94, as well as ∆E99 and ∆E94, contain in the calculation formulas the correction factors k (DIN99) k_L_, k_C_, and k_H_ (CIE94), respectively.
- The compression factor k (DIN99) is calculated by means of k_E_, which only has a value of 1 for reference (theoretical) conditions. Consequently, the authors determined the value of this factor experimentally in working conditions of a real service.
- The correction factors k_L_, k_C_, and k_H_ (CIE94) usually come from determinations made for the textile industry or for 3D printing and are assigned the value 1. Neither are these factors assigned values determined experimentally or calculated specifically for the automotive industry and in real working conditions. For CIE94, the authors have determined the K_L_ value in real conditions.
- In this study, DIN99 was chosen because in this space, contrary to CIE94, it is not necessary to determine the angle for calculating the color distance. Because it is designed for small and medium distances, in DIN99, the authors calculated the distances ∆E99.
- For quality assurance, the authors noticed that ∆E99 must have a value lower than 5. For some of the reading pairs for which ∆E99 < 5, the authors identified them as follows: 109–110, 113–114, 117–118, 138 -139, 140–143, 141–142, 144–145, and 146–147, and for these, the authors have identified the compression factor k.
- The identification of the k factor and the DIN99 space helped the authors to identify the dark shades. Shades of black are often found in the services where measurements were made. The compression factor was useful in identifying the shades of black, namely, black with a shade of green, black with a shade of red or black with a shade of brown (ochre).
- Authors optimized the K_L_ factor in CIE94 and obtained the value 1.0429 under the conditions K_C_ = 1 and K_H_ = 1.

## 4. Discussion

Because many pigments used for automotive refinishing are thermochromic to a certain extent, a temperature change of 10 °C can lead to color differences of up to ∆Eab = 0.6 for automotive coatings. So, color measurements must be performed in rooms where the temperature is controlled to remain constant within 1 degree. However, in car painting, this process takes place in large halls where such strict temperature control is not possible. Therefore, color is measured at temperatures that can vary from 15–30 °C depending on the seasons and local conditions. Our measurements were performed between June 2023 and July 2024, when the temperature varied between −6 degrees and 36 degrees.

The services where the authors performed the measurements are located in the Veneto region, the province of Venice at an altitude of 4m above sea level and 7 km from the Po Valley and the Adriatic Sea, respectively. The wettest period of the year lasts 3.6 months, from 1 June to 19 September, and during this period, the comfort level is oppressively unbearable, even intolerable at least 12% of the time. The month with the highest number of days with maximum humidity at Aeroporto di Venezia-Tessera [45] is July.

The year 2024 was a very rainy year, in which large quantities of fallen water and hail caused damage in all sectors of the automotive industry in Italy. Therefore, humidity is a factor that not only influences but also creates major problems if it is not taken into account in all services that paint with water-based paints.

Color measurements on strongly curved surfaces are problematic and should be avoided, which is impossible to achieve when car models stand out with extremely curved body shapes with super-specific lines such as those of the German manufacturer Porsche.

Also, another problem is that, in some car models, the adjacent parts are not parallel, but have a relatively small angle. For effect coatings, this can lead to a small but visible difference between these parts.

Future research directions are the construction of a virtual sensor that, taking data from a set of real sensors, is able to estimate the color variant closest to the objective color by adapting the correction factors and thus achieving efficiency in the color-identification process.

The color matching is very important in the opinion of experts from automotive painting in the following six situations, and also, in all of these cases, the optimization made through the authors’ study was essential and was validated by the improvements in color matching.

- “Critical colors such as Bianco Perlato are applied in a triple layer. In this case is very important the base support color, for which authors have made this optimization, and the two applied layers of the pearl, which changes its shade depending on the thickness of the applied layer are easy to apply”.
- “There are also pastel colors with weaker coverage. For example: black or red, and in this situation is also very useful the authors optimization”.
- “It is recommended to make several color samples to determine exactly the correct amount of paint applied to obtain uniformity, and with the authors optimization it can be reduced the quantity of used pain”.
- “The thickness of the applied layer is important, and with the authors optimization it can be reduced the thickness of the applied layer”.
- “It is important the case of transparent varnish that is applied at the end, because many transparent varnishes that have a yellow tint can change the effect of the color, and in this case the color differences are accentuated. An excess of varnish can lead also to errors in color identification, so, the author optimization is useful in this case”.
- “Because the air pressure variation during application influences the applied layer, the application pressure has an optimal value of 1.8–2 bar for the base, and a 2.5–3 bar for transparent depending on the manufacturer, and in this case the color differences are accentuated and the author optimization is very important”.

## 5. Conclusions

To make the color-identification process in car services more efficient, a set of conditions must be strictly observed. Authors performed measurements with spectrophotometers under the operating conditions of the services and identified the correction factors involved in two color spaces so that this study can quantify the algorithm for minimizing the color differences between the standard formula and the resulting formula. From the point of view of pigments and paints, color formulas have been continuously improved.

From the point of view of preparing the painting process, the body of a vehicle must be prepared so that the entire process is streamlined by reducing failures in identifying the correct color. The authors have identified the main factors involved which generated color differences.

When the outside temperature is above 30 degrees, theoretically, no color measurements should be performed, which is practically impossible because the repair process must be completed. This temperature effect is difficult to eliminate or avoid.

Parts and paint suppliers should agree on the use of the same standardized observation conditions and, more generally, the same visual-evaluation procedures.

This research used optimization techniques like the Generalized Reduced Gradient (GRG) method to fine-tune correction factors in color spaces such as CIELAB, DIN99, and CIE94. This authors’ research was carried out in real conditions, a car service, during an entire year, January 2023–January 2024, and was based on measurements made on more than 200 vehicles, all followed throughout the entire painting process. The main research result was the determination and the optimization of the correction factors in real conditions of painting in car services, and not the use of these factors resulting from other types of processes (textile industry, 3D printing, etc.), as they were used in other studies. The application of these factors calculated in an optimization process allowed for most of the analyzed vehicles to obtain a color where color differences were minimized between the situation when optimization for pain color was not applied and the result of painting after color optimization. This indicator, the color difference, is very difficult to quantify from an economic point of view, both for the service and for the customer, being an indicator that can be noticed most of the time only visually, but it is instead essential for customer satisfaction, especially in the case of cars with an above average price, such as those analyzed in our research. Both the beneficiary of the research results, services, as well as customers for more than 50 cars for which the authors have applied the optimization, declared themselves very satisfied with the practical results obtained based on the research carried out by the authors.

## Figures and Tables

**Table 1:** Experimental conditions: color, date, temperature, humidity and ∆V (result).

Data Set Readings	Color	Date	Temp. °C	Humidity	∆V
163	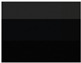	25 September 2023	19.44	73.5	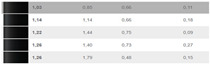
178	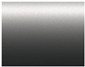	13 December 2023	27	83.1	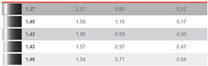
179	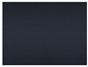	13 December 2023	27	83.1	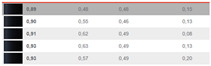
180	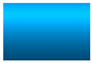	13 December 2023	27	83.1	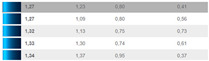
50	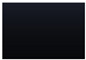	17 January 2024	22	85.1	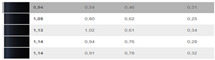
51	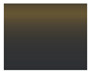	18 January 2024	22.2	92.5	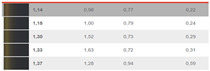
52	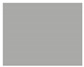	18 January 2024	22.2	92.5	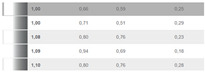
53	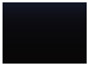	19 January 2024	21.7	87.2	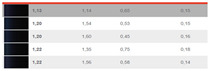
54	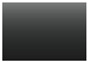	22 January 2024	21	96.6	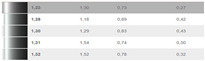

**Table 2:** Types of paint used in experiments—car model, color name and Color Code.

Data Set Readings	Color	Car Model	Color Name	Color Code
03	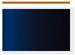	Honda	Blue	512
04	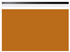	Toyota	Pollux Orange	304
05	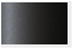	Volkswagen	Mangan Grey	LB7R
06	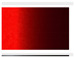	Mazda	Soul Red Crystal	B
07	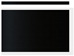	Mercedes	Obsidian Schwarz	198
15	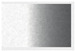	Yamaha	Grey Europe	711
16	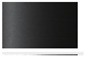	Ferrari	Nero	901C
17	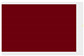	Honda	New Deep	R94
18	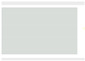	Porsche	Pure White/Weiss	149
19	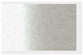	Mitsubishi	Silky White	CMW10013
20	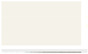	Mercedes	Polar Weiss	149

**Table 3:** Results after optimization; improvements represented in a visual manner for 6 cases.

Data Set Readings/Color	Visual and Cromax Detection of Initial Stage	Visual Detection After Optimization Results
37–41 Fiat Lancia Arancio Rosso Corallo 193	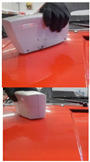	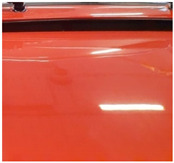
04 Toyota Orange Pollux 304	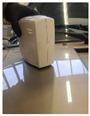	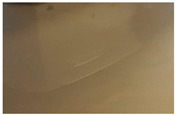
47 Audi R8 Nardo Grau LY7C	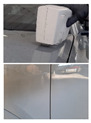	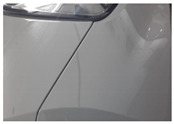
45–46 Porsche 2D8 Gruen	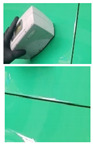	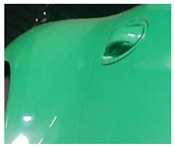
38–39 Porsche Carrera Beketts—Black 728	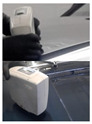	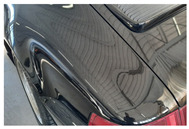
43–44 Porche L12L Pastel Gelb	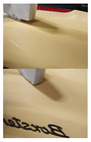	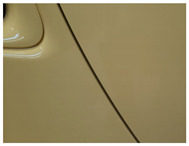

## Data Availability

The data presented in this study are available on request from the first author.
